# Outcome using selective hemihepatic vascular occlusion and Pringle maneuver for hepatic resection of liver cavernous hemangioma

**DOI:** 10.1186/s12957-015-0680-9

**Published:** 2015-09-04

**Authors:** Minghao Li, Chunyan Zhang, Tao Zhang, Liyun Wang, Yang Ding, Zhanxue Niu, Saiwu He, Zhiqi Yang

**Affiliations:** Hepatobiliary Surgery Department, Ningxia People’s Hospital, No.301, North Zhengyuan Street, Jinfeng District, Yinchuan, Ningxia 750002 China

**Keywords:** Liver cavernous hemangioma (LCH), Pringle maneuver (PM), Selective hemihepatic vascular occlusion (SHVO), Ischemia reperfusion injury

## Abstract

**Background:**

Though accumulated evidence proved the advantages of laparoscopic hepatectomy, bleeding still remains the most important challenge in clinical practice. Our study aimed to compare the outcomes of Pringle maneuver (PM) and selective hemihepatic vascular occlusion (SHVO) surgeries for patients with liver cavernous hemangioma (LCH).

**Methods:**

The SHVO (*n* = 26; mean age, 42) and PM (*n* = 78; mean age, 43) surgeries were performed in 104 LCH patients from January 2006 to January 2015. The intraoperative (bleeding, arterial pressure, oxyhemoglobin saturation, etc.) and postoperative parameters (anal exhaust time, complications, blood cell numbers, etc.) were measured and compared between the two groups. Liver function of all these patients was detected by blood test at 1-day preoperation, and at 1, 3, and 5 days postoperation.

**Results:**

Both of the two surgeries were successfully performed without any mortality. The intraoperative systolic arterial pressure and pulse in PM group were much higher than that in SHVO group (*P* < 0.01). The postoperative liver function parameters such as alanine transaminase (ALT), aspartate transaminase (AST), and total bilirubin (TBIL) increased much more in the PM group than that in the SHVO group compared with preoperation results (*P* < 0.05). However, there were no statistical differences in intraoperative bleeding, blood transfusion, hepatic inflow occlusion time, oxygen saturation occlusion, anal exhaust time and incidence of complications between the two groups (*P* > 0.05).

**Conclusions:**

SHVO is safer with less ischemia reperfusion injury than PM surgery for hemangioma resection on patients with LCH.

## Background

Liver cavernous hemangioma (LCH) is one of the most common benign tumors in liver, with incidence ranging from 5 to 20 % [1]. Most of the LCHs are small in size, and thus symptomatic in some patients require no treatment. However, some patients still need operative interventions because of its life-threatening complications [2].

Recently, several methods have been reported for LCH treatment, such as surgical resection [3], radiofrequency ablation [4], microwave coagulation [5], radiotherapy [6], etc. Among them, the surgical resection is reported to be the most effective treatment with good postoperative outcomes [3]. During surgical resection, bleeding is the most important challenge. Blood transfusions have been demonstrated to have a severe impact on long-term and short-term outcomes, that is called ischemia reperfusion injury [7]. Therefore, it is important to choose a suitable surgical resection approach with minimized blood loss for LCH treatment.

Pringle maneuver (PM) and selective hemihepatic vascular occlusion (SHVO) methods are two common methods for liver resection. PM is a technique of transient inflow occlusion that was first described by Pringle [8]. It is carried out by taping the hepatoduodenal ligament and then using a vascular clamp or a tourniquet until the hepatic arterial pulse disappears. Though the validity of PM is reported to reduce hemorrhage in liver resection, it usually induces ischemia reperfusion injury in many cases [9, 10]. In order to avoid the ischemia reperfusion injury, the SHVO technique which can allow normal blood supply at contralateral hemi-liver is proposed [11]. However, this technique will induce more bleeding from the other hemi-liver and will cause serious complications.

Though many studies have compared the outcome of these two techniques [12, 13], no consistent conclusion has achieved until now, especially for patients with LCH who need excision. A meta-analysis performed by Wang et al. [14] evaluated the outcomes of PM and SHVO, and the results suggested that SHVO did not offer more satisfying benefit than PM in patients who suffered from hepatic resection. However, a recent retrospective study compared the clinical outcomes of PM and SHVO in patients with liver tumor who need liver resection, and concluded that the SHVO using extra-Glissonian approach offers benefits of less blood loss and operative time, as well as better recovery in liver resection [15].

In this study, we retrospectively evaluated the outcome of the two techniques, PM and SHVO, aimed to find a better one for hemangioma excision for LCH patients.

## Methods

### Patients

From January 2006 to January 2015, 104 patients with LCH aged from 22 to 62 years (mean age: 42 years) who underwent hepatic resection in the Department of Hepatobiliary Surgery at the Ningxia Medical University were included in this study. All these patients were operated because the Child-Pugh score was class A, or the class B were adjusted to class A. They were performed SHVO or PM.

All the patients signed an informed consent and this study was approved by the local ethics research committee.

### Surgical procedure

SHVO:

There are two different approaches to achieve vascular occlusion in SHVO method, by liver parenchyma and by hilar plate.

The approach by liver parenchyma was described as follows: Firstly, a sharp knife was used to prick hole on liver capsule, then a right-angle forceps was inserted into the hole, and a blunt dissection was performed outside the Glisson’s sheath. In order to protect the portal vein and its small branches from damage, the forceps should mobilize the liver parenchyma towards the caudate with no resistance. At last, the sharp end of the forceps was passed through the junction of portal vein furcation and caudate lobe, and an 8-Fr catheter was introduced. The blood flow in the right and left half of the liver can be blocked when the catheter was crimpled during hepatic resection.

The approach by hilar plate was described as follows: A small incision was made in the base of the Glisson’s sheath. Then the right-angle forceps was gently passed through the plane between the hilar plate and the liver to dissect the hilar plate outside the Gilsson’s sheath, and the 8-Fr catheter was introduced. Finally, the vascular occlusion was achieved by tighten the catheter during hepatic resection.

PM:

The PM was performed by encircling the hepatoduodenal ligament with the 8-Fr catheter, and applying with 15-min clamping and 5-min release period.

### Intraoperative and postoperative evaluation

The intraoperative blood loss, blood transfusion, and occlusion time were measured. Besides, the peripheral blood pressure, pulse, and oxygen saturation occlusion were monitored. We also detected the postoperative anal exhaust time, the volume of abdominal drainage, diaphragmatic fluid infection, pleural effusion infection, biliary fistula, temperature, and peripheral blood. Liver function of all these patients was detected by blood test at 1 day preoperation, and at 1, 3, and 5 days postoperation.

### Statistical analysis

Data were expressed as mean ± standard deviation (SD). Comparison between groups was analyzed by *t* test. Repeated measures analysis of ANOVA was used to evaluate the quantitative variables, while enumeration data was determined by *χ*^2^ test. Significant difference was defined as *P* < 0.05.

## Results

### Demographic data of patients

Of the 104 patients, 26 (males/females, 4/22) with mean ages of 42.92 ± 11.47 years underwent SHVO surgery, and 78 (males/females, 24/54) with mean ages of 44.91 ± 8.52 years underwent PM surgery. In the two surgery groups, 2 (7.69 %) and 9 (11.54 %) patients were diagnosed as hepatitis, respectively. Of them, 2 (7.69 %) and 5 (64.10 %) patients were classified into Child-Pugh’s grade B, respectively; these patients had been adjusted to Child-Pugh’s grade A before surgery. The length of hospital stay was 16.81 ± 4.70 days and 16.56 ± 5.50 days in the SHVO group and the PM group, respectively. There were no significant differences between the two groups in gender, ages, hospital stay duration, and Child-Pugh’s classification (*P* > 0.05) (Table 1). There were 8 patients in the SHVO group and 21 patients in the PM group underwent additional cholecystectomy surgery.

**Table 1:** Comparison of general conditions of the patients between the two groups

Surgery groups	No.	Gender (male/female)	Age (year)	Hospital stay (day)	Hepatitis (yes/no)	Child-Pugh’s grade (A/B)
SHVO	26	4/22	42.92 ± 11.47	16.81 ± 4.70	2/24	24/2
PM	78	24/54	44.91 ± 8.52	16.56 ± 5.50	9/96	73/5
*t* (*χ* ^2^)		2.346	0.941	0.208	0.305	0.051
*P*	>0.05	>0.05	>0.05	>0.05	>0.05	>0.05

*SHVO* selective hemihepatic vascular occlusion, *PM* Pringle maneuver

### Intraoperative outcome

The intraoperative outcome of the two groups was listed in Table 2. Though the volume of blood loss and blood transfusion was more in the SHVO group than that in the PM group, the difference was insignificant (*P* > 0.05). Besides, the difference of oxyhemoglobin saturation in the two groups was also insignificant (*P* > 0.05). However, significant difference was found between the two groups in systolic arterial pressure and pulse (*P* < 0.01), indicating that the effect of hemodynamics caused by SHVO surgery was much less than that caused by PM surgery.

**Table 2:** The comparison of intraoperative outcome between the two groups

	SHVO	PM	*t*/*F*	*P*
Blood loss (mL)	1116.92 ± 925.33	855.13 ± 669.15	*t* = 1.562	>0.05
Blood transfusion (mL)	1015.38 ± 840.81	896.15 ± 915.16	*t* = 0.587	>0.05
Occlusion time (min)	29.12 ± 10.62	24.46 ± 10.30	*t* = 1.986	=0.05
Systolic arterial pressure (mmHg)			*F* = 7.878	<0.01
1-min before occlusion	121.65 ± 11.30	119.29 ± 11.56		
1-min after occlusion	122.15 ± 12.23	145.13 ± 17.20		
1-min after opening	119.04 ± 12.78	116.26 ± 13.37		
Pulse (time)			*F* = 9.208	<0.01
1-min before occlusion	79.19 ± 10.35	81.32 ± 10.64		
1-min after occlusion	81.77 ± 8.68	96.14 ± 14.36		
1-min after opening	79.04 ± 13.23	82.28 ± 12.59		
Oxyhemoglobin saturation (%)			*F* = 0.602	>0.05
1-min before occlusion	100.00 ± 0.00	99.99 ± 0.11		
1-min after occlusion	100.00 ± 0.00	100.00 ± 0.00		
1-min after opening	100.00 ± 0.00	99.98 ± 0.23		

### Postoperative outcome

No patients died during the perioperative period in the two surgery groups. In the PM group, 1 patient was found with diaphragmatic fluid infection, 2 were found with pleural effusion infection, 1 was detected with biliary fistula, 2 had raised body temperature, and 2 had raised blood cell numbers. The patients’ numbers of these complications in the SHVO group were 0, 2, 0, 1, 25, and 0, respectively. No statistical significance was found in these complications between SHVO and PM surgeries (*P* > 0.05). In addition, we also detected the anal exhaust time and the volume of abdominal drainage, and found no significant difference between the two groups as well (*P* > 0.05) (Table 3).

**Table 3:** The comparison of postoperative outcome between the two groups

	SHVO	PM	*t*/*χ* ^2^	*P*
Anal exhaust time (d)	3.15 ± 0.54	3.14 ± 0.42	*t* = 0.125	>0.05
Volume of abdominal drainage (mL)	312.00 ± 375.51	354.85 ± 358.33	*t* = 0.643	>0.05
Diaphragmatic fluid infection (yes/no)	0/26	1/77	*χ* ^2^ = 0.337	>0.05
Pleural effusion infection (yes/no)	2/24	2/76	*χ* ^2^ = 1.387	>0.05
Biliary fistula (yes/no)	0/26	1/77	*χ* ^2^ = 0.337	>0.05
Body temperature (increased/not increased)	1/25	2/76	*χ* ^2^ = 0.114	>0.05
Blood cell numbers (increased/back to normal)	0/26	2/76	*χ* ^2^ = 0.680	>0.05

The liver function parameters such as alanine transaminase (ALT), aspartate transaminase (AST), albumin (ALB), and total bilirubin (TBIL) are presented in Table 4. The results showed that all these liver function parameters were significantly different between the SHVO and PM groups after surgery. ALT, AST, and TBIL increased much more in the PM group than that in the SHVO group compared with preoperation results (*P* < 0.05). These results suggested that PM surgery can significantly induce ischemia reperfusion injury of the liver.

**Table 4:** The liver function parameters of the patients in the two groups before and after surgery

Liver function	SHVO				PM				*F*	*P*
	1-day before surgery	1-day after surgery	3-day after surgery	5-day after surgery	1-day before surgery	1-day after surgery	3-day after surgery	5-day after surgery		
ALT (IU/L)	21.58 ± 19.66	462.77 ± 293.19	321.52 ± 209.53	138.83 ± 91.72	20.77 ± 30.92	650.46 ± 421.77	449.00 ± 412.24	236.59 ± 199.01	4.078	<0.05
AST (IU/L)	19.42 ± 4.93	506.18 ± 370.87	237.26 ± 199.95	66.08 ± 53.11	22.20 ± 23.24	683.62 ± 427.03	368.05 ± 346.49	124.40 ± 160.56	4.250	<0.05
ALB (g/L)	39.54 ± 3.47	31.88 ± 5.66	34.33 ± 3.71	40.45 ± 3.50	39.61 ± 5.11	27.31 ± 5.32	30.06 ± 4.00	35.25 ± 4.60	27.071	<0.01
TBIL (μmol/L)	11.70 ± 5.37	15.50 ± 7.07	16.90 ± 7.35	16.91 ± 3.83	12.02 ± 5.00	18.15 ± 6.98	19.78 ± 6.88	19.67 ± 6.60	4.733	<0.05

*ALT* alanine transaminase, *AST* aspartate transaminase, *ALB* albumin, *TBIL* total bilirubin

## Discussion

This study evaluated the intraoperative and postoperative outcome of SHVO and PM surgeries for hemangioma excision in LCH patients. The results showed that the intraoperative systolic arterial pressure and pulse in the PM group were much higher than that in the SHVO group, indicating that the effect of hemodynamics caused by SHVO surgery was much less than that caused by PM surgery. In addition, the postoperative liver function parameters such as ALT, AST, and TBIL increased much more in the PM group than that in the SHVO group compared with preoperation results, suggesting that PM surgery can significantly induce ischemia reperfusion injury of the liver.

Liver is an organ that is sensitive to ischemia and anoxia. A loss of blood supply will result in reducing oxygen supply to the liver and finally cause ischemia injury. When the blood is transfused into the ischemic liver, it will affect the activity of oxygen-dependent cells and cause impairment of organ function. That is called ischemia reperfusion injury [9].

PM is a common surgical practice and gained wild acceptance due to its simple and time-saving advantages. During PM surgery, intermittent clamping of hepatic inflow other than continuous clamping is proved safer [16]. Usually, 120 min is considered to be the safe upper limit [17]. However, this technique is reported to cause severe ischemia reperfusion injury even though it performed with ischemic preconditioning [18]. The vascular occlusion, especially long time continuous occlusion, will result in unstable hemodynamics, increased arterial pressure, and decreased cardiac index [16]. In this study, though we performed the PM surgery with 15-min clamping and 5-min release period, we also found that the patients in the PM group showed much more increased intraoperative systolic arterial pressure and pulse than those in the SHVO group. A recent study compared the short-term outcome of patients who underwent hepatectomy with intermittent clamping (ranging from 60 to 120 min) with those having a clamping time more than 120 min, and suggested that both of the two clamping frequencies are safe for patients [19]. We believe that the clamping frequency is associated with the blood loss and ischemia reperfusion injury during surgery, thus, further research should be performed to clarify these associations.

SHVO is a technique that can reduce the ischemia reperfusion injury by blocking blood supply of tumor location at a hemihepatic portion and allowing normal blood supply at the contralateral hemi-liver. SHVO surgery over 60 min is possible and sufficient for hepatectomy [20]. In the present study, the recovery of liver function parameters in the SHVO group was as fast as that in the PM group after surgery. Besides, the recovery in the SHVO group had much less effect of ischemia reperfusion injury than PM surgery. Our result was consistent with a previous study reported by Ni et al., who compared the outcomes between SHVO and PM surgeries [21]. The results of this study showed that the PM surgery was associated with much higher postoperative ALT and AST levels than SHVO surgery. However, the study did not compare the intraoperative outcomes between the two methods. Our results of the intraoperative outcomes showed a significant difference between the two groups in systolic arterial pressure and pulse, indicating that the effect of hemodynamics caused by SHVO surgery was much less than that caused by PM surgery. Therefore, more evidence from our results demonstrated that SHVO is an efficient method in recovery of liver function without any short-term complications after surgery for LCH patients who need hemangioma excision. However, this technique is a complicated and difficult surgery. Surgeons should have experience in dissecting the porta hepatis and lowering hilar plate to protect the vessels and bile ducts from injury [10]. Besides, before surgery, surgeons should be aware of the position and size of the lesions, as well as the preoperative liver function index.

There are several limitations that exist in our study. Firstly, the patient size is small and most patients are women. As we know, LCH is a liver disease that are most frequently seen in women, and most of the patients need no surgery [22, 23]. That is the reason why only 104 patients were included in this study during 9 years. Besides, a surgeon’s experience and preference remain the possible factors in deciding the most appropriate methods to be used in clinical practice. Therefore, more patients and multicenter trials are needed to acquire more reliable data in future studies. Secondly, we just compared the short-term postoperative outcome of the SHVO and PM surgeries, thus long-term follow-up should be performed to completely analyze the feasibility of the two methods.

## Conclusions

In summary, our study compared the short-term outcomes of SHVO and PM surgeries on LHC patients and demonstrated that SHVO is safer with less ischemia reperfusion injury than PM surgery. However, further studies with more patients and long-term follow-up durations are required to compare the safety and effectiveness of the two techniques.

1. The efficacy and safety of PM and SHVO surgeries were evaluated.
2. Arterial pressure and pulse in PM group were much higher than that in SHVO group.
3. Liver function parameters increased much more in PM group than that in SHVO group.
4. SHVO is safer with less ischemia reperfusion injury than PM surgery.

## Abbreviations

ALB: albumin
ALT: alanine transaminase
AST: aspartate transaminase
LCH: liver cavernous hemangioma
PM: Pringle maneuver
SD: standard deviation
SHVO: selective hemihepatic vascular occlusion
TBIL: total bilirubin
